# *Penicillium citrinum* UFV1 *β*-glucosidases: purification, characterization, and application for biomass saccharification

**DOI:** 10.1186/s13068-018-1226-5

**Published:** 2018-08-20

**Authors:** Samara G. da Costa, Olinto Liparini Pereira, André Teixeira-Ferreira, Richard Hemmi Valente, Sebastião T. de Rezende, Valéria M. Guimarães, Fernando Ariel Genta

**Affiliations:** 1 Laboratory of Enzymatic Technology, Department of Biochemistry and Molecular Biology, Federal University of Viçosa, Viçosa, MG CEP3657000 Brazil; 2 Laboratory of Insect Biochemistry and Physiology, Oswaldo Cruz Institute, FIOCRUZ, Rio de Janeiro, Av Brasil 4365, Pav. Leônidas Deane, Room 207, Manguinhos, RJ CEP21040360 Brazil; 3 Laboratory of Seed Pathology and Post-Harvest, Department of Phytopathology, Federal University of Viçosa, Viçosa, MG CEP36570-000 Brazil; 4 Laboratory of Toxinology, Oswaldo Cruz Institute, FIOCRUZ, Rio de Janeiro, RJ CEP21040360 Brazil; 5 National Institute of Science and Technology for Molecular Entomology, Rio de Janeiro, RJ Brazil

**Keywords:** *β*-Glucosidase, *Penicillium citrinum*, Biomass hydrolysis, Cellulase, Synergism, Inhibition, Subsite analysis

## Abstract

**Background:**

*β*-Glucosidases are components of the cellulase system, a family of enzymes that hydrolyze the *β*-1,4 linkages of cellulose. These proteins have been extensively studied due to the possibility of their use in various biotechnological processes. They have different affinities for substrates (depending on their source) and their activities can be used for saccharification of different types of biomass. In this context, the properties and the synergistic capacity of *β*-glucosidases from different organisms, to supplement the available commercial cellulase cocktails, need a comprehensive evaluation.

**Results:**

Two *β*-glucosidases belonging to GH3 family were secreted by *Penicillium citrinum* UFV. PcβGlu1 (241 kDa) and PcβGlu2 (95 kDa) presented acidic and thermo-tolerant characteristics. PcβGlu1 showed Michaelis–Menten kinetics for all substrates tested with *K*_m_ values ranging from 0.09 ± 0.01 (laminarin) to 1.7 ± 0.1 mM (cellobiose, C2) and *k*_cat_ values ranging from 0.143 ± 0.005 (laminarin) to 8.0 ± 0.2 s^−1^ (laminaribiose, Lb). PcβGlu2 showed substrate inhibition for 4-methylumbelliferyl-*β*-d-glucopyranoside (MUβGlu), *p*-nitrophenyl-*β*-d-glucopyranoside (pNPβGlu), cellodextrins (C3, C4, and C5), *N*-octil-*β*-d-glucopyranoside, and laminaribiose, with *K*_m_ values ranging from 0.014 ± 0.001 (MUβGlu) to 0.64 ± 0.06 mM (C2) and *k*_cat_ values ranging from 0.49 ± 0.01 (gentiobiose) to 1.5 ± 0.2 s^−1^ (C4). Inhibition constants (*K*_i_) for PcβGlu2 substrate inhibition ranged from 0.69 ± 0.07 (MUβGlu) to 10 ± 1 mM (Lb). Glucose and cellobiose are competitive inhibitors of PcβGlu1 and PcβGlu2 when pNPβGlu is used as a substrate. For PcβGlu1 inhibition, *K*_i_ = 1.89 ± 0.08 mM (glucose) and *K*_i_ = 3.8 ± 0.1 mM (cellobiose); for PcβGlu2, *K*_i_ = 0.83 ± 0.05 mM (glucose) and *K*_i_ = 0.95 ± 0.07 mM (cellobiose). The enzymes were tested for saccharification of different biomasses, individually or supplementing a *Trichoderma reesei* commercial cellulose preparation. PcβGlu2 was able to hydrolyze banana pseudostem and coconut fiber with the same efficiency as the *T. reesei* cocktail, showing significant synergistic properties with *T. reesei* enzymes in the hydrolysis of these alternative biomasses.

**Conclusions:**

The *β*-glucosidases from *P. citrinum* UFV1 present different enzymatic properties from each other and might have potential application in several biotechnological processes, such as hydrolysis of different types of biomass.

**Electronic supplementary material:**

The online version of this article (10.1186/s13068-018-1226-5) contains supplementary material, which is available to authorized users.

## Background

*β*-Glucosidases (EC 3.2.1.21) constitute a group of well-characterized enzymes, displaying several functions, of biological and industrial relevance. These enzymes catalyze the hydrolysis from the nonreducing termini of *β*-glycosidic bonds present in short-chain oligosaccharides (containing 2–6 monosaccharides), alkyl- and aryl *β*-d-glucosides [1, 2]. They are also involved in transglycosylation reactions of *β*-glucosidic linkages of glucose conjugates. *β*-Glucosidases are widely distributed in living organisms, and the affinity of these enzymes for a particular substrate is dependent on their physiological function, location, and the nature of the enzyme source [3].

*β*-Glucosidases are also a component of the cellulase system, a family of enzymes that hydrolyze the *β*-1,4 linkages of cellulose, which presents three categories of enzymatic components: (1) endo-glucanases (EC 3.2.1.4), which act on the cellulose chain catalyzing the random cleavage of internal bonds to yield glucose and cello-oligosaccharides; (2) cellobiohydrolases (EC 3.2.1.91), which release cellobiose from the reducing and nonreducing ends of the polysaccharide, and (3) *β*-glucosidases (cellobiase, EC 3.2.1.21), which release glucose from cello-oligosaccharides [4, 5]. Because both endoglucanase and exoglucanase activities are usually inhibited by cellobiose and short cello-oligosaccharides, *β*-glucosidases are responsible for the rate-limiting step of the whole cellulolytic process in vitro [6, 7].

These enzymes constitute a significant group among glycoside hydrolases, and the possibility of their use in various biotechnological processes has been intensely explored. Considering industrial applications, *β*-glucosidases are currently used in: production of biodegradable nonionic surfactants and other compounds [8]; synthesis of diverse oligosaccharides, glycoconjugates, alkyl- and amino-glycosides [5]; detoxification of cassava [9]; removal of cyanogenic glucosides from sorghum malt used in the production of African beer [10]; enzymatic release of aroma compounds from glucosidic precursors present in fruit juices during winemaking [11]; enhancement of tea extracts aroma [12], among others. These enzymes can play a critical role in the generation of potentially sustainable energy sources (e.g., glucose, ethanol, hydrogen, and methane) from biomass conversion [13].

The production of ethanol from renewable sources has gained attention in the last decade due to the concern over depleting fossil fuel and the impact on the environment. Different bio-renewable materials have arisen as potential sources for alternative fuel production [14], such as sugars, starch, and lignocellulosic material. The lignocellulosic biomass is abundantly available and is a ubiquitous source of energy; most importantly, it does not compete with food production and animal feed. Cellulosic sources as corn stover, sugarcane bagasse, rice, and wheat straws are the most promising sources to be uses as substrates for bioethanol production [15–18]. Moreover, this other lignocellulosic materials can be obtained from industrial wastes, wood, and agricultural residues.

Commercial cellulolytic preparations for biomass hydrolysis, such as Celluclast ^®^ (Novo Nordisk), Acellerase^®^ (Sigma-Aldrich), and the newly developed Cellic^®^ CTec2 and Cellic^®^ CTec3 (Novozymes), incorporate fungal glycosyl hydrolases in their composition, especially from *Trichoderma reesei*. One of the most studied fungi for cellulose hydrolysis is *Trichoderma reesei,* as it secretes a cellulase mixture composed mainly of exoglucanases and endo-glucanases (> 92%), albeit just a few *β*-glucosidases [19]. Hence, an efficient biomass conversion of commercial preparations using *T. reesei* is dependent on the supplementation with exogenous *β*-glucosidase. High amounts of cellobiose are produced by exo- and endo-glucanases, but *β*-glucosidases undertake the rate-limiting step by hydrolyzing cellobiose to glucose [20]. In short, these are crucial enzymes for the process of biomass bioconversion, and finding a *β*-glucosidase capable of efficiently supplementing these cocktails remains a major bottleneck, since cellobiose is a potent inhibitor of cellulase activities.

A few fungal strains are known to be efficient producers of *β*-glucosidases. Among them, some filamentous thermophilic fungi are good sources of *β*-glucosidases with high thermal stability, a most desirable property for industrial purposes [21]. Several works have demonstrated the production of *β*-glucosidases from different fungal species, including *Chrysoporthe cubensis* [22], *Talaromyces leycettanus* [20], *Acremonium thermophilum* (AtBG3), and *Thermoascus aurantiacus* (TaBG3) [23], aiming supplementation to enhance the saccharification efficiency of cellulosic materials.

In this study, we isolated a cellulolytic fungus*, Penicillium citrinum* UFV1, from sugarcane bagasse. Production of an endoglucanase, xylanase, and *β*-galactanase [24–26] from *P. citrinum* has been reported and *β*-glucosidase activity from *Penicillium citrinum* YS40-5 was already described [4]. However, the detailed kinetic properties and the possible application of these enzymes for the saccharification process of natural biomass were not yet explored.

Exploiting the fact that these enzymes have different affinities for substrates, depending on enzyme source, the present study describes (a) the production, purification, and biochemical characterization of two *β*-glucosidases from *P. citrinum* UFV1; (b) these enzymes’ application on the saccharification process of sugar cane bagasse and two other alternative biomasses, coconut fiber and colloidal banana pseudostem; and (c) their synergistic effect to the *Trichoderma reesei* cellulase system (the most common commercial source of cellulases).

## Results

### Enzyme production

**Fig. 1:**
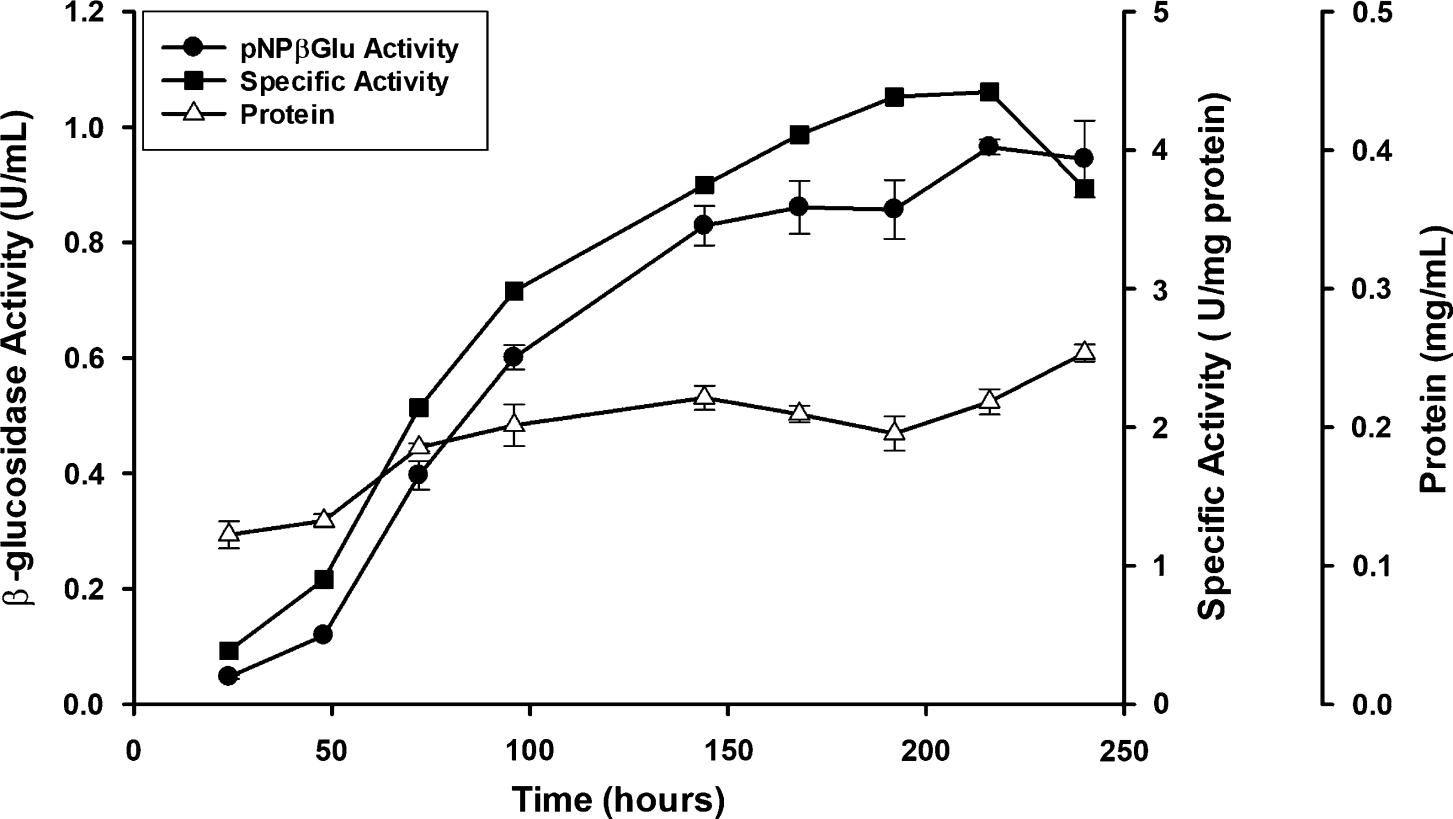
β-Glucosidase activity in the secretion of *Penicillium citrinum* UFV1 cultivated in submerged culture containing wheat bran as a carbon source. Activity was determined using pNPβGlu substrate. Error bars indicate the standard error of the mean of 3 experimental replicates

First, we investigated the potential of *P. citrinum* UFV1 to produce *β*-glucosidases growing on low-cost biomass. The fungus was grown for 10 days under submerged culture using wheat bran as a carbon source and *β*-glucosidase activity in the soluble fraction was monitored (Fig. 1). *β*-Glucosidase activity increased until 8–9 days of fermentation when maximal specific activities were observed (4.3 ± 0.3 - 4.4 ± 0.1 U mg^−1^). Other glycoside hydrolases were produced in these conditions, especially xylanase and cellulase (Additional file 1: Table S1). A fermentation lasting 8 days was established as the standard protocol for enzyme production, and subsequent purification of *P. citrinum* UFV1 *β*-glucosidases.

### Purification and sequence identification by mass spectrometry

**Fig. 2:**
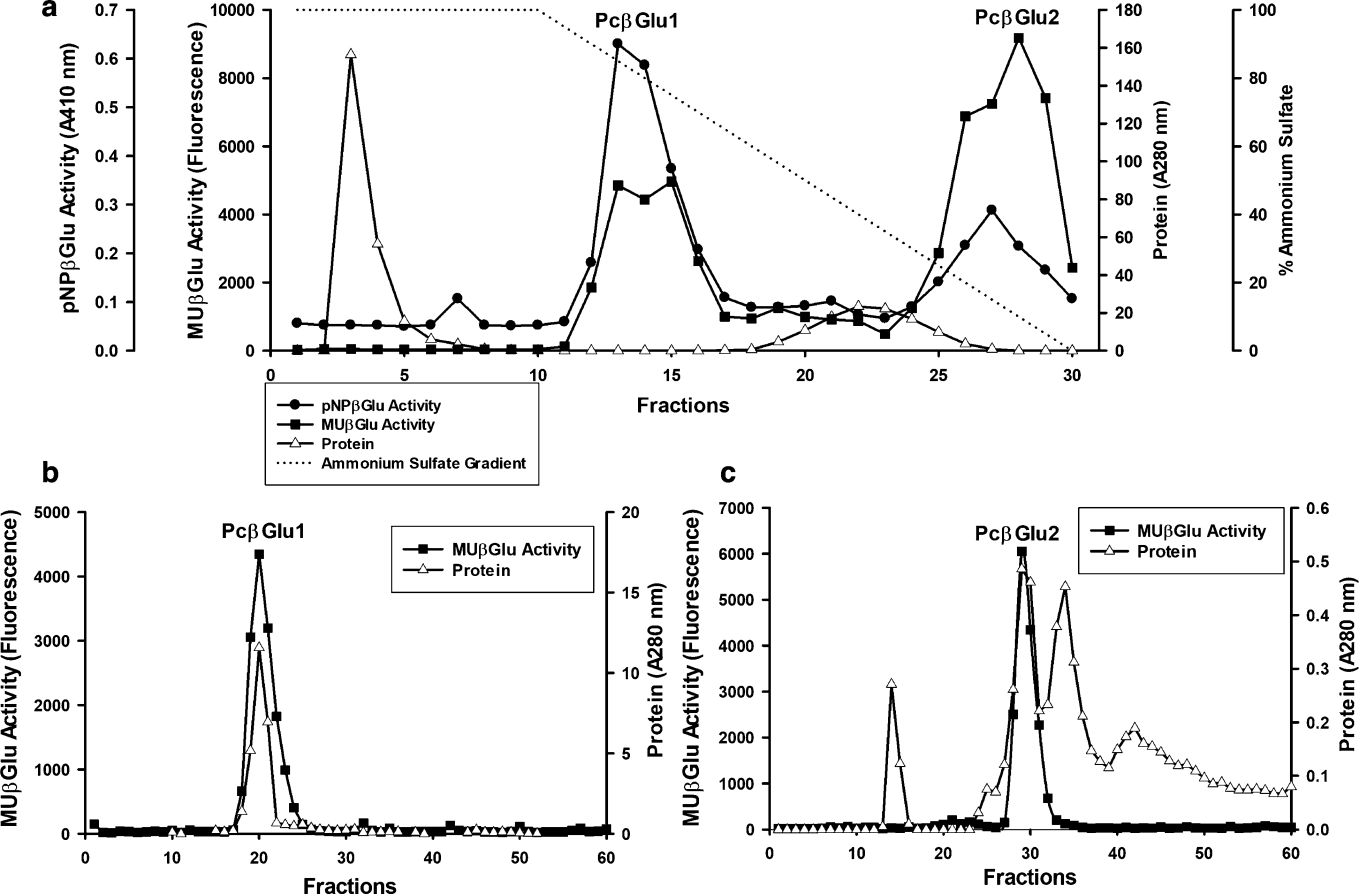
Chromatographic profile obtained after β-glucosidase purification. **a** Pellet from ammonium sulfate precipitation was resuspended and applied onto a phenyl sepharose column (AKTA PRIME, GE Healthcare Biosciences). Purified PcβGlu1—fractions 12–16. PcβGlu2—fractions 25–30. Activity was determined using pNPβGlu and MUβGlu substrates. **b** PcβGlu1 fractions from phenyl sepharose were pooled and applied onto a Superdex 200 column for molecular mass determination. **c** PcβGlu2 fractions from phenyl sepharose were pooled and applied onto a Superdex 200 column fractions for further purification and molecular mass determination. Purified PcβGlu2—fractions 29–32. Activity was measured with MUβGlu substrate

*Penicillium citrinum* UFV1 *β*-glucosidases were successfully purified in few steps (Table 1). The supernatant culture was subjected to 50% ammonium sulfate precipitation and the remaining supernatant, after centrifugation, was applied to a hydrophobic interaction chromatography. The activity was eluted in two peaks after chromatography, PcβGlu1 and PcβGlu2 (Fig. 2a). PcβGlu1 was more active on pNPβGlu than on MUβGlu, while PcβGlu2 was more active on MUβGlu (Fig. 2a). After this step, PcβGlu1 was obtained as a homogeneous protein (see below). For further purification of PcβGlu2 and molecular mass measurements, PcβGlu1 and PcβGlu2 were applied onto a gel filtration Superdex S200 column (AKTA Purifier); each active enzyme displayed its own retention time (Fig. 2b, c). PcβGlu1 was purified 3.9-fold with a yield of about 9.9%, while PcβGlu2 was purified 3.4-fold with a yield of 3.2% (Table 1).

**Table 1:** Typical parameters for purification of *β*-glucosidases from *Penicillium citrinum* UFV1

Purification step	Total activity (mU)	Total protein (mg)	Specific activity (U/mg of protein)	Purification (fold)	Yield (%)
Crude extract	3464.1	34.2	101.4	1.0	100.0
Ammonium sulfate precipitation	2666.0	12.9	205.9	2.0	77.0
Phenyl sepharose					
PcβGlu1	341.6	0.9	398.3	3.9	9.9
PcβGlu2	547.2	1.6	338.1	3.3	15.8
Superdex 200					
PcβGlu2	111.1	0.3	349.2	3.4	3.2

**Fig. 3:**
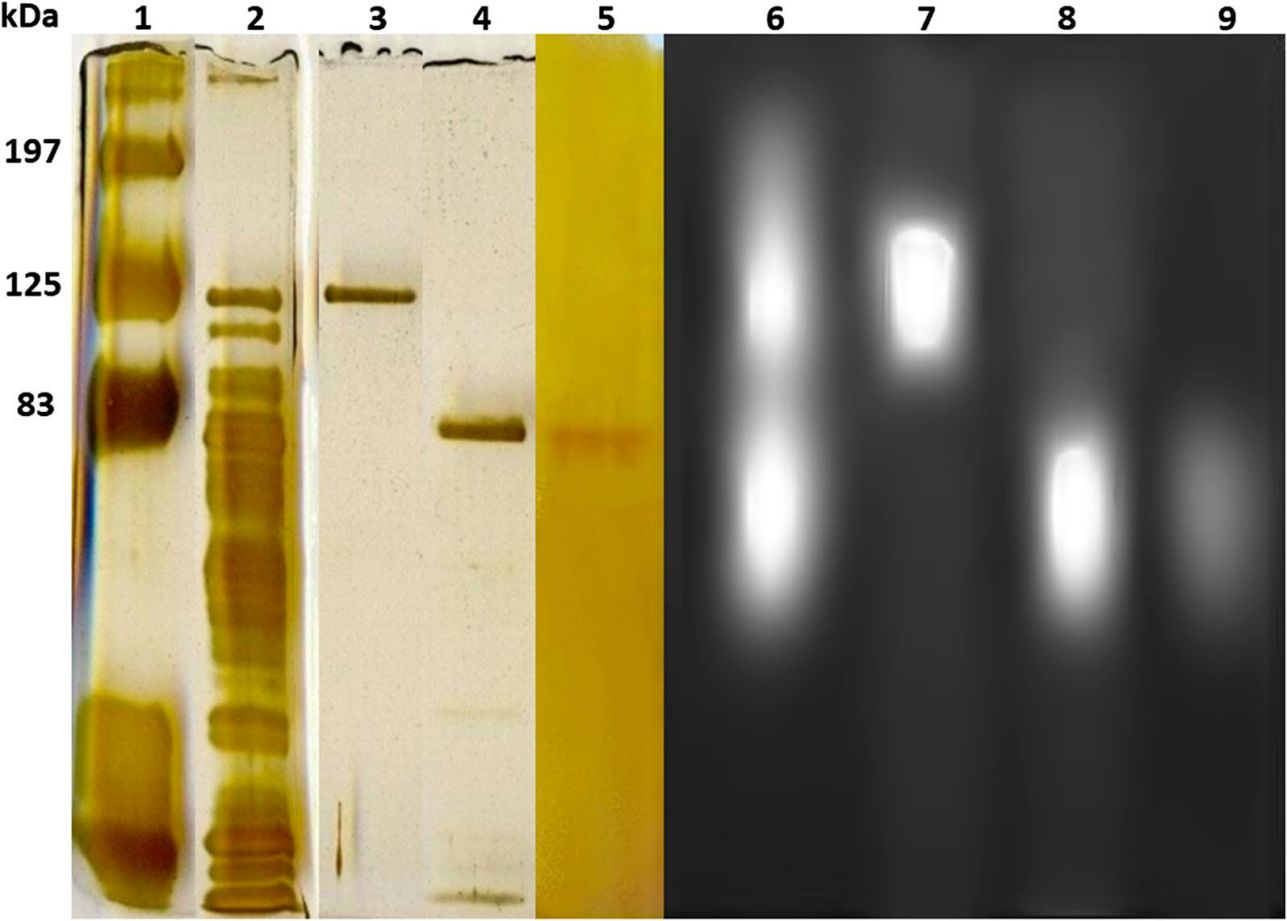
SDS-PAGE and in-gel assays after PAGE of PcβGlu1 and PcβGlu2. Lanes 1–5: SDS-PAGE (silver staining); lanes 6–9: in-gel assays using MUβGlu as a substrate. 1 Molecular mass markers. 2 and 6 *P. citrinum* crude extract. 3 and 7 PcβGlu1 after phenyl sepharose (see Fig. 2a). 4 and 8 PcβGlu2 after phenyl sepharose (see Fig. 2a). 5 and 9 PcβGlu2 after chromatography in Superdex 200 column (see Fig. 2c)

Purified PcβGlu1 and PcβGlu2 were revealed as unique bands when submitted either to PAGE (detection by in-gel assays with MUβGlu) or SDS-PAGE (protein staining) indicating the homogeneity of the preparations (Fig. 3, lanes 2–5). Besides, PcβGlu1 and PcβGlu2 activity bands corresponded to the two regions of activity observed in the soluble fraction of *P. citrinum* UFV1 (Fig. 3, lanes 6–9).

The molecular mass of PcβGlu1 was estimated as 134 kDa by SDS-PAGE (Fig. 3, lanes 3) and 258 kDa by gel filtration chromatography (Fig. 2b). Hence, it was assumed that native PcβGlu1 is a homodimeric protein. Analysis of PcβGlu2 using the same techniques and conditions revealed a molecular mass of 93 kDa (SDS-PAGE) and 89 kDa (gel filtration chromatography), suggesting that the native form of this protein is monomeric.

Purified PcβGlu2 was very unstable after removal of the ammonium sulfate from the chromatographic buffer (data not shown). The addition of substrates, sugars, detergents, and proteins was tested for stabilization at − 20 and 4 °C (Additional file 2: Table S2). The best stabilizer was bovine albumin serum (BSA) at 1 mg mL^−1^, which maintained PcβGlu2 activity for at least 1 week at 4 °C.

Purified PcβGlu1 was characterized by shotgun mass spectrometry coupled to data analysis with the PEAKS Studio software (http://www.bioinfor.com/peaks-studio/) against a comprehensive Uniprot “Penicillium” database (338,849 entries). Identified peptides matched several protein groups, with a major contribution of sequences from the glycoside hydrolase family 3 (GH3) (Additional file 3: Table S3). It is important to notice that none of the other hits obtained corresponded to protein families with *β*-glucosidase activity (E.C. 3.2.1.21), with some of these sequences matching pseudogenes or protein entries with no associated catalytic activity information. Unfortunately, analysis of purified Pcβglu2 did not yield significant results, mostly due to sample contamination with keratin (data not shown). Hence, we present here the data that we had previously obtained using the heterogeneous fraction obtained following hydrophobic interaction chromatography (Fig. 2a), which was enriched for this enzyme. As expected, the results showed a high diversity of protein contaminants, but we observed a sizable contribution of protein sequence hits belonging to the GH3 family (Additional file 4: Table S4). Despite the identification of an array of glycoside hydrolases from different families, all the protein sequences identified in this sample that do not belong to GH3 lack both beta-glucosidase activity and the catalytic active site residues (as the proton donor and nucleophile) that are typical for this enzyme group (Additional file 3: Table S3, Additional file 4: Table S4). In this respect, mass spectrometry data indicate that both PcβGlu1 and PcβGlu2 might belong to the GH3 group of enzymes, with a more confident identification for the first enzyme.

### Effect of pH, temperature, and metal ions on PcβGlu1 and PcβGlu2

**Fig. 4:**
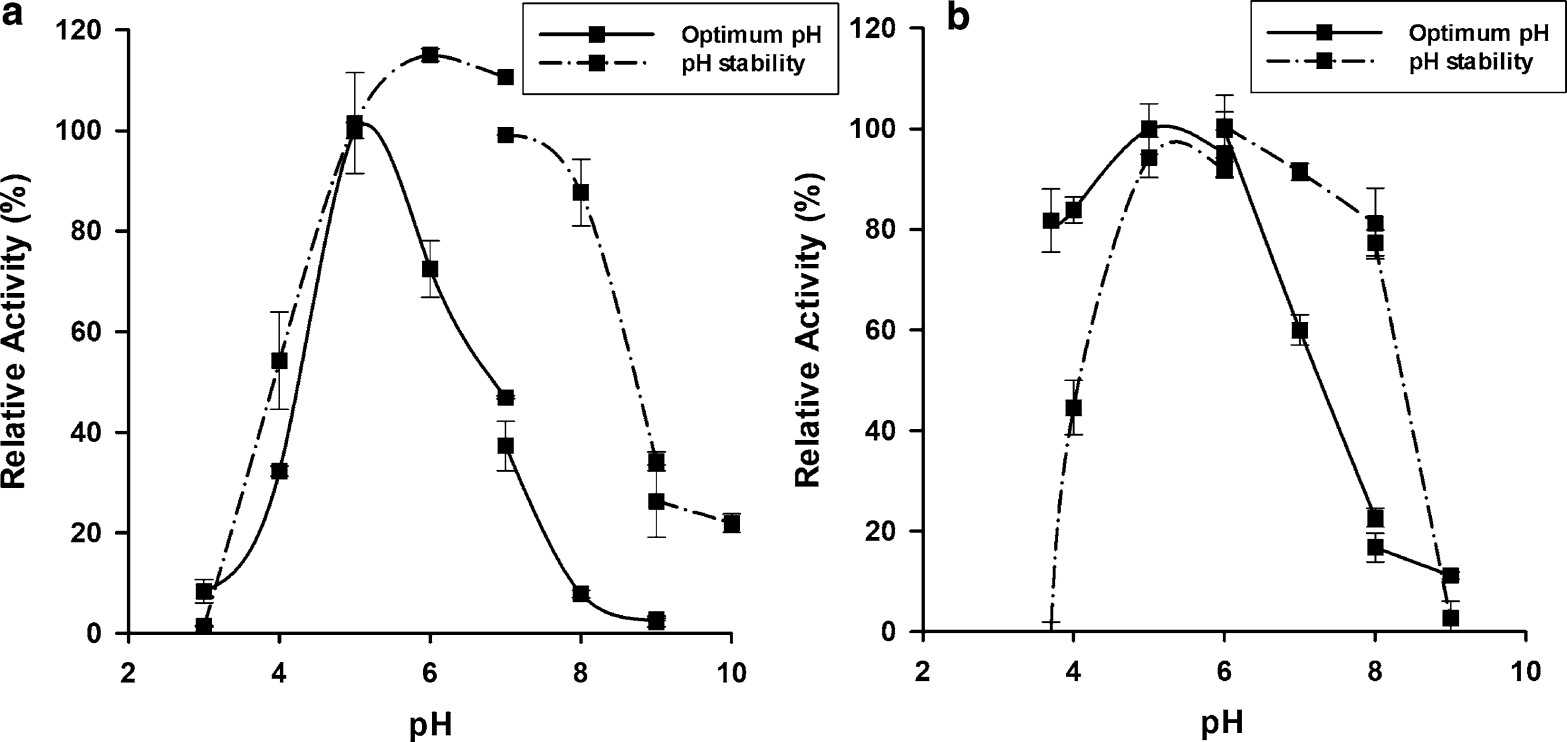
Effect of pH on PcβGlu1 and PcβGlu2 activities. Enzymes were incubated in different buffers from pH 3 to 10 at 40 °C. For stability tests, the enzymes were pre-incubated for 4 h and residual activity was measured at the optimum pH. Activities were measured with MUβGlu as a substrate. **a** PcβGlu1. **b** PcβGlu2

**Fig. 5:**
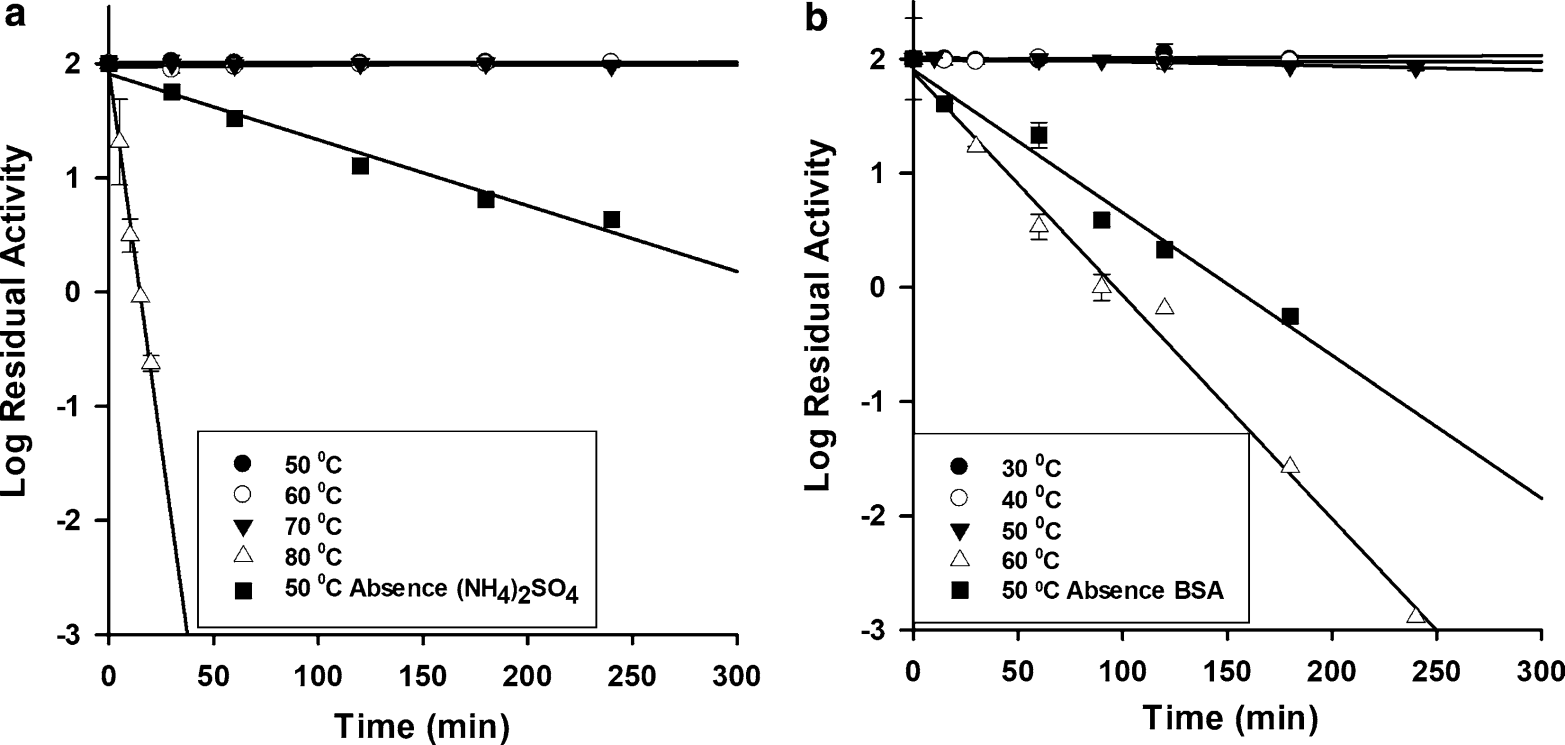
Effect of temperature on PcβGlu1 and PcβGlu2 activities. Enzymes were pre-incubated at different temperatures until 4 h. Residual activity was measured using MUβGlu as a substrate. **a** PcβGlu1 and **b** PcβGlu2. Linear regression was used to calculate ***k***_***d***_ values and half-life

**Fig. 6:**
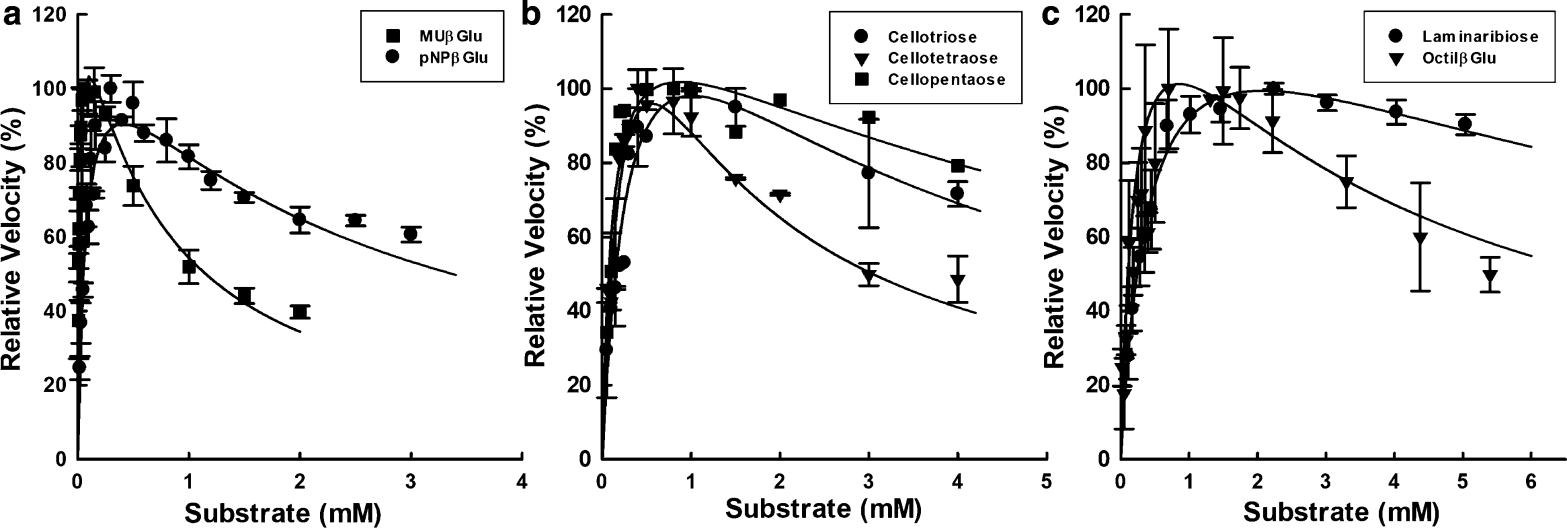
Effect of substrate concentration on PcβGlu2 velocity. Theoretical curves (lines) were determined using kinetic parameters from Table 5. **a** 4-Methylumbelliferyl β-d-glucopyranoside and *ρ*-nitrophenyl-glucopyranoside. **b** cellotriose, cellotetraose, and cellopentaose. **c** laminaribiose and octyl-*β*-glucopyranoside

Effect of pH on the *β*-glucosidases activities was determined with MUβGlu as a substrate at 40 °C and different pH (3–10). For pH stability evaluation, the enzymes were incubated at 40 °C for 4 h at various pH before residual activity determination. Both enzymes were stable for 4 h at 40 °C at pH range 5–8 (Fig. 4). Purified PcβGlu1 and PcβGlu2 showed maximal activity at pH 5 (Fig. 4a) and 5–6 (Fig. 4b), respectively. PcβGlu1 was more susceptible to pH change than PcβGlu2.

Thermal stability of *β*-glucosidases was determined by incubating the enzymes for 4 h at 30–80 °C before residual activity determination. PcβGlu1 was stable in the presence of ammonium sulfate during 4 h at 50, 60, and 70 °C; at 80 °C, the enzyme was inactivated after 15 min (Fig. 5a). In the absence of ammonium sulfate, PcβGlu1 showed a half-life of 51 ± 2 min and *k*_*d*_ of 2.3 × 10^−4^ ± 1 × 10^−5^ s^−1^ at 50 °C.

PcβGlu2 was stable in the presence of BSA (1 mg mL^−1^) for 4 h at 30 and 40 °C (Fig. 5b). For 50 °C and 60 °C, the calculated half-life is demonstrated in Table 2. At 70 °C, the enzyme was completely inactivated after 4 min (data not show). In the absence of BSA, PcβGlu2 showed a half-life of 36 ± 6 min and *k*_*d*_ of 3.4 × 10^−4^ ± 5 × 10^−5^ s^−1^ at 30 °C. Values of *k*_*d*_ and half-life for PcβGlu1 and PcβGlu2 are demonstrated in Table 2; it is important to stress that *k*_*d*_ values are inversely proportional to the protein’s thermostability, that is, the higher the *k*_*d*_, the lower will be the protein stability. Hence, the data confirm (Table 2) that PcβGlu1 was more thermostable than PcβGlu2.

**Table 2:** Thermoinactivation constant (*k*_*d*_) and half-life (*T*_1/2_) for PcβGlu1 in the presence of ammonium sulfate and for PcβGlu2 in the presence of BSA when incubated at different temperatures

	Incubation temperature			
	50 °C	60 °C	70 °C	80 °C
*k*_*d*_ PcβGlu1	–	–	1.4 × 10^−6^ ± 5 × 10^−7^	4.4 × 10^−3^ ± 8 × 10^−4^
*k*_*d*_ PcβGlu2	1.4 × 10^−5^ ± 2 × 10^−6^	7.3 × 10^−4^ ± 3 × 10^−5^	–	–
*T*_1/2_ PcβGlu1	–	–	9000 ± 900	2.6 ± 0.5
*T*_1/2_ PcβGlu2	800 ± 100	16 ± 1	–	–
*k*_*d*_ (s^−1^)	*T*_1/2_ (min)			

As shown in Fig. 5, the increase in temperature resulted in a progressive inactivation of both enzymes, PcβGlu1 being more thermo-tolerant than PcβGlu2. The thermostability of PcβGlu1 is increased by the presence of ammonium sulfate. At low concentrations, this salt can stabilize proteins by non-specific electrostatic interactions which depend on the ionic strength of the medium [27]. PcβGlu1 and PcβGlu2 activity loss at high temperatures followed first-order kinetics, which indicated an irreversible inactivation by a monomolecular process [28].

Effects of various ions on *β*-glucosidases activity were investigated by pre-incubating the enzymes for 30 min at 40 °C in the presence of these ions before residual activity determination. Purified PcβGlu1 and PcβGlu2 activities were highly affected in the presence of 100 mM Fe^3+^, Ba^2+^, and Pb^2+^ (Table 3). PcβGlu2 was also affected by Cu^2+^ and Hg^+^, whereas PcβGlu1 showed higher residual activities of 29.6 ± 0.4 and 56 ± 3% after incubation with Cu^2+^ and Hg^+^, respectively. No significant inhibition occurred in the presence of Ca^2+^, Zn^2+^, Co^2+^, and Mn^2+^ for PcβGlu1. Nevertheless, PcβGlu2 activity was affected especially by Zn^2+^ incubation. Small activation of PcβGlu1 and inhibition of PcβGlu2 was observed in the presence of Mg^2+^.

**Table 3:** Effect of metal ions on PcβGlu1 and PcβGlu2 activities

	Residual activity (%)	
	PcβGlu1	PcβGlu2
Control	100 ± 5	100 ± 2
NaCl	106 ± 6	101 ± 3
NaNO_3_	101 ± 5	100 ± 10
KCl	110 ± 2	90 ± 10
CuSO_4_	29.6 ± 0.4	1.68 ± 0.03
BaCl_2_	13.2 ± 0.2	0.8 ± 0.2
MgCl_2_	110 ± 10	63 ± 3
CaCl_2_	88 ± 1	86 ± 5
ZnCl_2_	91 ± 2	22 ± 3
HgCl	56 ± 3	0.55 ± 0.01
Pb(NO_3_)_2_	1.2 ± 0.1	3 ± 1
NH_4_Cl	108.4 ± 0.2	100 ± 20
MnCl_2_	72 ± 2	60 ± 10
CoCl_2_	78 ± 1	72 ± 9
FeCl_3_	0.32 ± 0.05	0.54 ± 0.05

Enzymes were incubated at 40 °C for 30 min in the presence of ions and residual activity was measured with MUβGlu as a substrate

### Kinetic parameters and subsite analysis

Kinetic parameters of purified *β*-glucosidases were determined using series of concentrations of different substrates. PcβGlu1 and PcβGlu2 showed broad substrate specificity, hydrolyzing pNPβGlu, MUβGlu, OctylβGlu, cellobiose, cellotriose, cellotetraose, cellopentaose, gentiobiose, laminaribiose, and laminarin (Tables 4, 5). In contrast, both enzymes showed no hydrolytic activity against pNPβGal, MUβMan, Avicel^®^, or CMC (data not shown). The configuration of the anomeric carbon of released glucose molecules by PcβGlu1 and PcβGlu2 is *β* (data not show), as determined by the glucose oxidase method (see “Methods”). This result suggested that the reaction catalyzed by these enzymes occured by the classical mechanism of retention of configuration of the anomeric carbon.

**Table 4:** Kinetic parameters of purified PcβGlu

Substrate	*K*_m_ (mM)	*k*_cat_ (s^−1^)	*k*_cat_*/K*_m_ (s^−1^ mM^−1^)
pNPβGlu	0.28 ± 0.01	20.1 ± 0.4	71.9 ± 2.7
MUβGlu	0.24 ± 0.01	13.1 ± 0.5	54.5 ± 2.5
Gentiobiose	0.38 ± 0.04	9.1 ± 0.4	24.0 ± 2.6
Cellobiose	1.7 ± 0.1	9.2 ± 0.4	5.4 ± 0.3
Cellotriose	0.8 ± 0.1	11.0 ± 0.8	13.7 ± 1.8
Cellotetraose	0.42 ± 0.05	11.8 ± 0.9	28.0 ± 3.5
Cellopentaose	0.25 ± 0.02	11.1 ± 0.7	44.5 ± 3.8
Laminaribiose	0.33 ± 0.04	9.8 ± 0.6	29.6 ± 3.7
Octyl-*β*-glucopyranoside	0.54 ± 0.03	7.2 ± 0.3	13.4 ± 0.8
Laminarin	0.09 ± 0.01	0.53 ± 0.04	5.9 ± 0.7

**Table 5:** Kinetic parameters of purified PcβGlu2

Substrate	*K*_m_ (mM)	*k*_cat_ (s^−1^)	*k*_cat_*/K*_m_ (s^−1^ mM^−1^)	*K*_i_ (mM)
pNPβGlu	0.072 ± 0.007	1.36 ± 0.03	18.9 ± 1.9	2.4 ± 0.2
MUβGlu	0.014 ± 0.002	1.51 ± 0.06	105.2 ± 11.8	0.7 ± 0.1
Gentiobiose	0.62 ± 0.07	0.61 ± 0.02	1.0 ± 0.1	–
Cellobiose	0.64 ± 0.06	0.35 ± 0.01	0.55 ± 0.05	–
Cellotriose	0.31 ± 0.08	2.09 ± 0.04	6.7 ± 1.9	3.3 ± 1.2
Cellotetraose	0.28 ± 0.07	3.2 ± 0.4	11.2 ± 3.4	1.1 ± 0.3
Cellopentaose	0.07 ± 0.01	1.6 ± 0.3	22.3 ± 3.2	4.3 ± 0.8
Laminaribiose	0.42 ± 0.03	1.55 ± 0.06	3.7 ± 0.3	10.2 ± 1.3
Octyl-*β*-glucopyranoside	0.19 ± 0.03	0.26 ± 0.02	1.4 ± 0.2	4.2 ± 0.7
Laminarin	0.027 ± 0.001	1.20 ± 0.02	44.3 ± 1.8	–

Kinetic parameters for the hydrolysis of various substrates by purified PcβGlu1 and PcβGlu2 are presented in Tables 4 and 5, respectively.

The best substrates for PcβGlu1 were pNPβGlu and MUβGlu, with higher catalytic efficiencies values. PcβGlu2 hydrolyzed MUβGlu more efficiently than PcβGlu1, which preferred pNPβGlu as a substrate. Differences in catalytic efficiencies of PcβGlu1 against these substrates were due mainly to variations in the *k*_cat_ value, whereas for Pcβglu2, these differences resulted from different binding affinities of the enzyme to the substrates (e.g., lower *K*_m_ for MUβGlu).

For cellodextrins, the highest catalytic efficiency was identified for cellopentaose hydrolysis by PcβGlu1 and PcβGlu2. PcβGlu1 hydrolyzes laminaribiose, gentiobiose, and octilβGlu with similar catalytic efficiencies, with a lower efficiency on laminarin (5.9 ± 0.9 s^−1^ mM^−1^). PcβGlu2 showed a high catalytic efficiency against laminarin (44.3 ± 1.8 s^−1^ mM^−1^), mainly due to a high binding affinity (lower *K*_m_) to this substrate. PcβGlu2 showed very low catalytic efficiencies for laminaribiose, octylβGlu, and gentiobiose.

PcβGlu1 showed classical Michaelis–Menten kinetics for all substrates. However, PcβGlu2 showed classical Michaelis–Menten kinetics when acting on gentiobiose, cellobiose, and laminarin hydrolysis, and showed enzyme inhibition by high concentrations of the substrates pNPβGlu, MUβGlu, laminaribiose, cellotriose, cellotetraose, cellopentaose, and octylβGlu (Fig. 6a–c). It is interesting to note the results obtained for cellodextrins, where cellotetraose showed the lowest *K*_i_ value (1.1 ± 0.3 mM), inhibiting PcβGlu2 at a lower concentration than cellotriose and cellopentaose (Table 5).

**Fig. 7:**
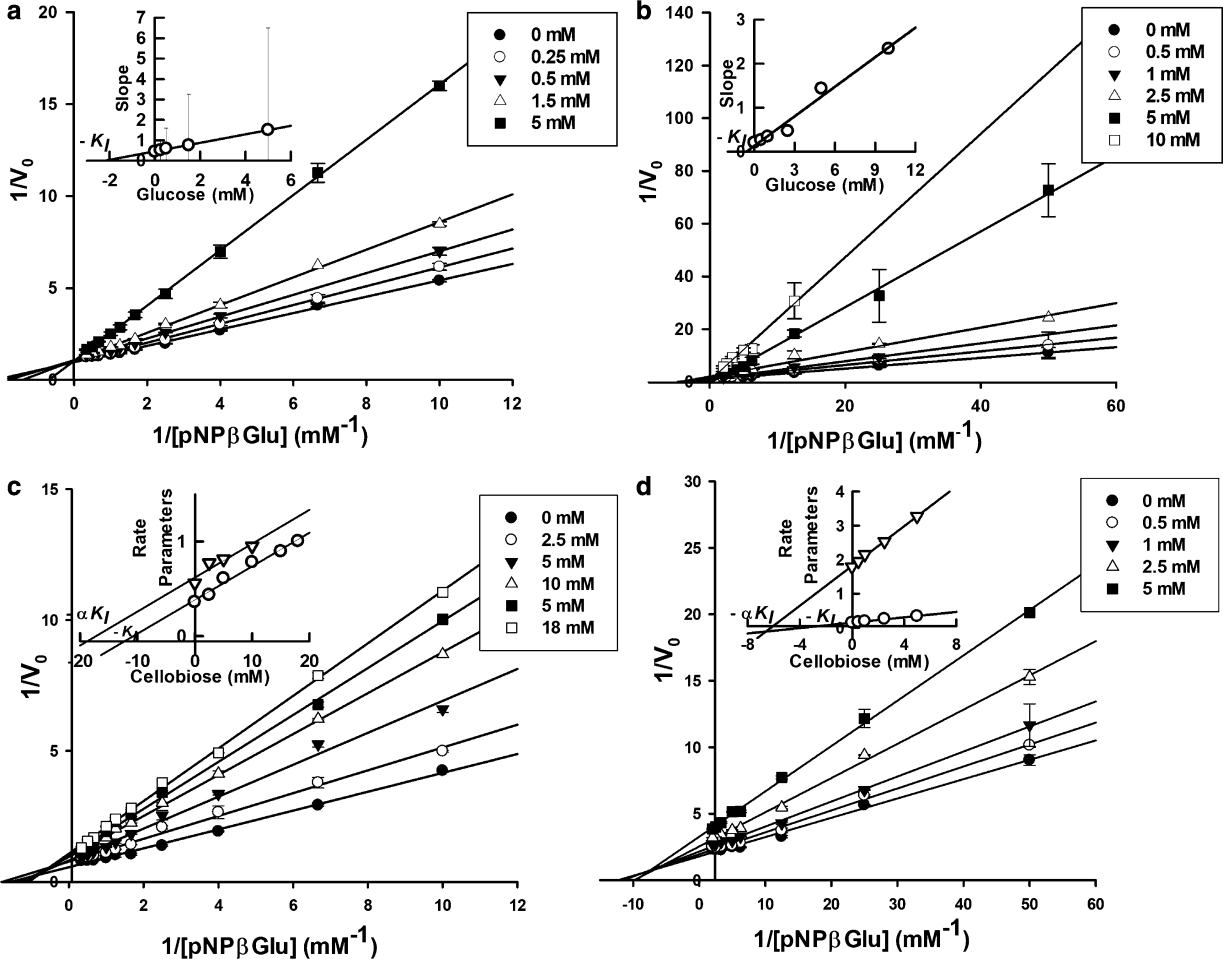
Graphic analysis of the inhibition of *P. citrinum β*-glucosidases. Lineweaver–Burk plot of initial velocity versus various fixed substrate concentrations showing inhibitory effects of glucose on pNPβGlu hydrolysis by **a** PcβGlu1 and **b** PcβGlu2. *Inset:* slope plot from the same data. Lineweaver–Burk plot of initial velocity versus various fixed substrate concentrations showing inhibitory effects of cellobiose on pNPβGlu hydrolysis by **c** PcβGlu1 and **d** PcβGlu2. Insets: slope and intercept plots from the same data

Glucose competitively inhibited the hydrolysis of pNPβGlu by PcβGlu1 and PcβGlu2, with *K*_i_ values of 1.89 ± 0.08 and 0.88 ± 0.08 mM, respectively (Fig. 7a, b). Cellobiose inhibited the hydrolysis of pNPβGlu by PcβGlu1 and PcβGlu2 in a mixed-type noncompetitive mode, with *K*_IE_ = 10.7 ± 0.1 mM and *K*_IES_ = 18.7 mM for PcβGlu1 (Fig. 7c) and *K*_IE_ = 3.74 ± 0.07 mM and *K*_IES_ = 6.2 mM for PcβGlu2 (Fig. 7d). These values reflect a higher affinity binding to the inhibitor for the free enzyme (*K*_IE_) than for the enzyme–substrate complex (*K*_IES_).

**Fig. 8:**
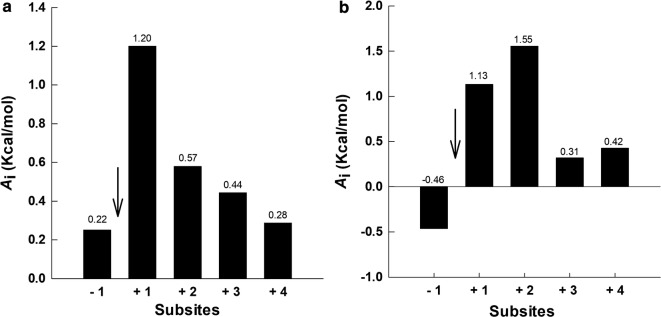
Histograms showing subsite affinities for glucosyl binding of *P. citrinum β*-glucosidases. The arrow indicates the position of the catalytic site. **a** Subsite affinities for PcβGlu1. **b** Subsite affinities for PcβGlu2

There were at least five subsites for binding glucosyl residues in the active sites of PcβGlu1 and PcβGlu2, numbered − 1, + 1, + 2, + 3, and + 4 (Fig. 8). The substrate is hydrolyzed between subsites − 1 and + 1. The subsite + 1 and subsite + 2 had higher affinities for glucosyl residues than the other subsites in both enzymes (Fig. 8), but the profile observed demonstrated differences in the catalytic site of these enzymes. The intrinsic catalytic constant was determined to be 49.2 s^−1^ for PcβGlu1 and 9.4 s^−1^ for PcβGlu2. The maximum deviation within 14% between experimental and theoretical rate parameters confirmed the validity of the equations and the calculated subsite affinities (Additional file 5: Table S5).

### Biomass saccharification

**Fig. 9:**
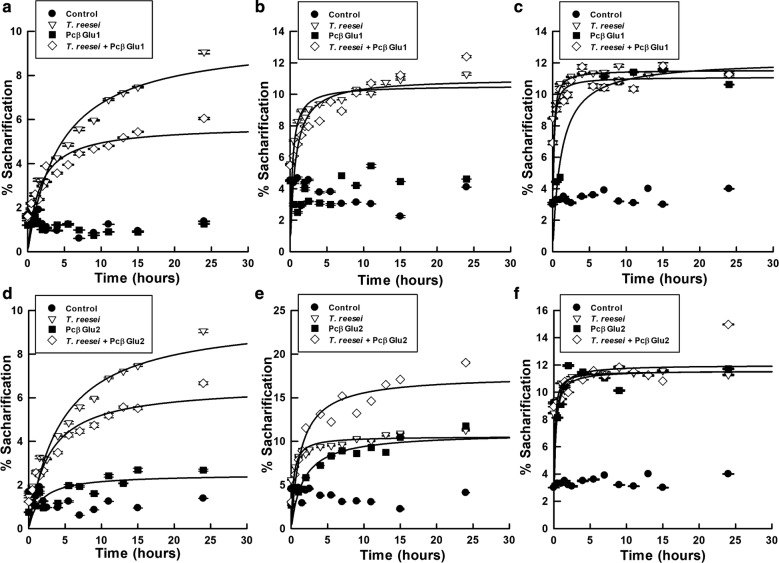
Time course of glucose production in experiments of enzymatic saccharification of colloidal biomasses (2.5% (w/v)) by *T. reesei* cellulases and PcβGlu1 or PcβGlu2. **a**, **d** Hydrolysis of sugar cane. **b**, **e** Hydrolysis of banana pseudostem. **c**, **f** Hydrolysis of coconut fiber. **a**–**c** Supplementation of *T. reesei* cellulases with PcβGlu1, and **d**–**f** show supplementation with PcβGlu2. Ten CBU/g of biomass were used for PcβGlu1 and PcβGlu2, while for *T. reesei* cellulases 7 CBU/g were used

We supplemented the cellulases from *T. reesei* with purified PcβGlu1 and PcβGlu2 from *P. citrinum* UFV1 for hydrolysis of colloidal preparations of sugarcane bagasse, banana leaf, and coconut fiber with minimal pre-treatment [30]. The enzymes of *P. citrinum* UFV1 were able to hydrolyze coconut fiber with the same capacity as *T. reesei* cellulases (Fig. 9c, f), and the quantity of reducing sugars released from this source of biomass after 24 h of saccharification was increased when *T. reesei* cocktail was supplemented with PcβGlu2 (Table 6).

**Table 6:** Effect of *Penicillium citrinum* and *Thricoderma reesei* enzymes on sugar cane bagasse, banana leaf, and coconut fiber hydrolysis

	Sugar cane bagasse				Banana leaf				Coconut fiber			
	V_0_ (µmol/h)^c^	T_50%_ (h)^d^	% maximum hydrolysis	Reducing sugars (mM)	V_0_ (µmol/h)^c^	T_50%_ (h)^d^	% maximum hydrolysis	Reducing sugars (mM)	V_0_ (µmol/h)^c^	T_50%_ (h)^d^	% maximum hydrolysis	Reducing sugars (mM)
*T. reesei*	0.05 ± 0.01	4.4 ± 1.1	9.7 ± 0.8^a^	14.1 ± 1.2^a^	0.09 ± 0.02	0.3 ± 0.2	10.6 ± 0.7^a^	11.9 ± 0.8^a^	0.07 ± 0.03	0.1 ± 0.1	11.5 ± 0.9^a^	6.2 ± 0.3^a^
PcβGlu1	–	–	–	–	–	–	–	–	0.06 ± 0.02	4.5 ± 2.4	12.2 ± 2.8^a^	4.2 ± 0.1^a^
PcβGlu2	0.03 ± 0.01	1.6 ± 0.8	2.5 ± 0.3^b^	4.4 ± 0.7^b^	0.059 ± 0.009	1.2 ± 0.4	10.5 ± 0.7^a^	10.2 ± 0.1^a^	0.07 ± 0.02	0.1 ± 0.2	10.8 ± 1.2^a^	5.8 ± 0.2^a^
*T. reesei *+ PcβGlu1	0.04 ± 0.02	1.6 ± 0.5	5.7 ± 0.5^a^	11.5 ± 0.9^a^	0.042 ± 0.002	0.6 ± 0.3	11 ± 1^a^	10.8 ± 0.7^a^	0.07 ± 0.04	0.2 ± 0.2	8.0 ± 0.7^a^	5.1 ± 0.9^a^
*T. reesei* + PcβGlu2	0.06 ± 0.02	2.6 ± 0.7	6.6 ± 0.5^a^	13.0 ± 0.9^a^	0.12 ± 0.02	1.3 ± 0.3	18 ± 1^b^	20.0 ± 1.2^b^	0.05 ± 0.03	0.2 ± 0.2	12.0 ± 1.2^a^	10.5 ± 0.3^b^

Same letters indicate no significant difference, *p* < 0.05

^c^Initial velocity hydrolysis

^d^Time to get 50% of maximum hydrolysis under test conditions

However, while PcβGlu2 was able to hydrolyze sugarcane bagasse (2.5% maximum hydrolysis) and banana pseudostem (10.5% maximum hydrolysis) (Fig. 9d, e), PcβGlu1 had no activity on these substrates (Fig. 9a, b). When using banana pseudostem as a substrate, Pcβglu2 had the same efficiency as *T. reesei* enzymes and demonstrated the ability to supplement the cellulases of *T. reesei* in banana pseudostem hydrolysis. The maximum percentage of hydrolysis of this substrate, after 24 h of saccharification, increased from 10.6 to 18.0% with the addition of PcβGlu2, and the amount of reducing sugars generated increased from 11.9 to 20.0 mM (Fig. 9e and Table 6).

It is interesting to note that the *T. reesei* cocktail displayed different reducing sugar production capacities depending on the type of biomasses. For instance, this enzyme sources had better activity on sugarcane bagasse than on coconut fiber.

The results demonstrated that PcβGlu1 and PcβGlu2 have different abilities to hydrolyze colloidal substrates (Table 6) and that PcβGlu2 is the most promising for supplementation of *T. reesei* cellulases aiming the hydrolysis of biomass.

## Discussion

Filamentous fungi are known as good producers of *β*-glucosidases and a large number of *β*-glucosidases from family GH1 and GH3 have been purified and characterized from these organisms [31]. The ability of fungi to grow on low-cost substrates makes them potential sources of enzymes for industrial applications. The production of cellulases and hemicellulases by filamentous fungi is strongly affected by culture conditions. The carbon source plays a central role in enzyme production, and cellulolytic enzymes are better induced by carbohydrates or their derivatives [32]. In this study, two *β*-glucosidases with different properties (PcβGlu1 and Pcβglu2) were produced and purified from *Penicillium citrinum* UFV1 cultivated in submerged culture, using wheat bran as carbon source.

The results for *β*-glucosidase production by *P. citrinum* UFV1 cultivation in submerged fermentation (SMF), containing wheat bran as a carbon source, have the same order of magnitude than those obtained by well-known cellulolytic species, such as *Aspergillus* spp., *Penicillium* spp., and *Trichoderma* spp., which are employed for the industrial production of cellulases [33–35]. Several investigations with different cellulolytic microorganisms have emphasized the advantages of wheat bran as a substrate to obtain high cellulase productions [33, 36]. Besides, wheat bran is a byproduct of industries and a carbon source broadly available.

The production of *β*-glucosidases isoforms by filamentous fungi has been previously reported [5, 37, 38] and the multiplicity of *β*-glucosidases can be attributed to the presence of multiple genes or due to differential post-transcriptional modifications [39]. As we demonstrated in this work, two different *β*-glucosidases were identified for *Penicillium citrinum* in the conditions studied. The purified PcβGlu1 and PcβGlu2 had different molecular masses, being characterized as homodimeric and monomeric proteins, respectively. Following peptide identification by mass spectrometry, in conjunction with available literature data and purified PcβGlu1/PcβGlu2 enzymatic characterization (this work), both enzymes were tentatively classified as belonging to the GH3 family. Dimeric *β*-glucosidases were described for *Paecilomyces thermophile* [21] and *Humicola insolens* [37], showing native molecular masses of about 200 kDa. Values ranging from 40 to 250 kDa were reported for *β*-glucosidases from different fungal sources, with wide variation concerning their molecular mass and the homogeneity of subunits, mainly due to post-translational modifications, since some *β*-glucosidases are highly glycosylated [3].

The structure of GH3 members includes an N-terminal (*α*/*β*)8 barrel followed by a C-terminal (*α*/*β*)6 sandwich domain. In general, the catalytic nucleophile is an aspartate found in the conserved motif VMSDW located in the TIM barrel domain and a glutamate acts as a proton donor in the catalytic site [40]. The active site is situated in the pocket at the interface between the two domains [1, 3, 41]. Additional domains can be found in some GH3 structures like the C-terminal fibronectin type III domain in Bgl3B from *T. neapolitana* and also in KmBglI from *K. marxianus*. The latter also has a PA14 domain inserted into the (*α*/*β*)6 sandwich domain that influences carbohydrate-binding [42, 43].

The classification of PcβGlu1 and PcβGlu2 in the GH3 family is consistent with the results showing that a retention mechanism exists and that Cu^2+^ and Zn^2+^ were inhibitory to the enzymatic activity of these proteins, which further supports the notion that basic (Arg, Lys, and His) and acidic (Asp and Glu) amino acids can be present in the catalytic domains [44]. The fact that these enzymes are differently affected by these metal ions demonstrates differences in their catalytic domains.

The determinations of enzymatic characteristics showed that PcβGlu1 and PcβGlu2 are acidic *β*-glucosidases with stability in a pH range from 5 to 8 and that PcβGlu1 is more thermostable than PcβGlu2. PcβGlu1 showed an estimated half-life of 150 h at 70 °C and PcβGlu2 of 13 h at 50 °C. The thermostability of these enzymes is an interesting characteristic for application in various biotechnological processes. In general, *β*-glucosidases produced by different fungi species have an acidic characteristic with highest activities between pH 4 and 6 [34, 46, 47]; the thermal stability of these enzymes is comparable to other thermo-tolerant *β*-glucosidases reported [2, 4, 20, 47]. However, it is important to highlight that our results suggest that PcβGlu1 and Pc βGlu2 require specific conditions for stabilization, such moderate ionic strength (obtained with ammonium sulfate in our experiments) or moderate protein concentrations (mimicked with BSA in our tests). Both restraints should probably not be impeditive their application in industry, as these conditions are normally obtained in general biomass degradation reactions.

Based on substrate specificity, *β*-glucosidases have been classified as (i) aryl *β*-glucosidases, which act on aryl-glucosides, (ii) true cellobiases, which hydrolyze cellobiose to release glucose, and (iii) broad substrate specificity *β*-glucosidases, which act on a wide spectrum of substrates [3]. PcβGlu1 and PcβGlu2 from *P. citrinum* UFV1 showed significant activity for both aryl-glucosides and cello-oligosaccharides, indicating that they belong to the group of broad substrate specificity *β*-glucosidases. Moreover, PcβGlu2 hydrolyzed laminarin with high efficiency, indicating that this *β*-glucosidase can display an additional exo-*β*-1,3-glucanase activity. Both glucosidases hydrolyzed *β*-1,3, *β*-1,4 and *β*-1,6 linked disaccharides. Broad specificity and exo-*β*-glucanase activity were already described for other GH3 glucosidases [45, 48, 49].

Kinetic parameters for various *β*-glucosidases have been determined using different substrates in the presence and the absence of glucose and *K*_i_ and *K*_m_ values have a lot of variation [23, 50]. PcβGlu1 and PcβGlu2 *K*_*m*_ values decreased with the increasing length of the substrates cellobiose, cellotriose, cellotetraose, and cellopentaose, suggesting that these enzymes have at least five subsites which bind glucosyl residues. An extended binding site also suggests a role for these enzymes in the degradation of oligosaccharides beyond the hydrolysis of cellobiose and other disaccharides, reinforcing their broad specificity. As the intrinsic catalytic constant calculated for PcβGlu1 (49.2 s^−1^) and PcβGlu2 (9.43 s^−1^) are higher than the catalytic constants determined for cellodextrins (Table 6), non-productive complexes are expected to be formed [29].

For Pcβglu2, some substrates did not follow classical Michaelis–Menten kinetics, because enzyme was inhibited by high concentrations of substrate (Fig. 6). This fact can be explained by transglycosylation reactions or by the binding of a second molecule of the substrate in the active site (substrate inhibition). Different works have found that for GH3 *β*-glucosidases with a retaining mechanism, the reduced hydrolytic activity relies at least in part on the occurrence of transglycosylation [38, 45, 51, 52]. Coherently, PcβGlu1 and PcβGlu2 are retaining enzymes, and *β*-glucosidases that hydrolyze substrates by double displacement mechanisms are classified in families GH1, GH3, GH5, GH16, and GH30 [53]; this kind of mechanism also permitting enzymes to transglycosylate.

Glucose and cellobiose inhibited PcβGlu1 and PcβGlu2 in competitive and mixed noncompetitive modes of inhibition, respectively. These types of inhibition suggest a second binding site for cellobiose in the active site of these enzymes near the active site, as the *K*_i_ values determined for cellobiose are higher than its *K*_m_ values. This second binding site could correspond to peripheral subsites in the active site, but more evidence is necessary to corroborate this hypothesis.

The *β*-glucosidases studied in this work showed different specificities and characteristics and they are potential sources to be used in different biotechnological applications. The rate and cost limiting steps in the production of biofuels from biomass in the conversion of polysaccharides to glucose are well known [20]. Consequently, *β*-glucosidases acting synergically with other enzymes could improve the results of biomass transformation making the process viable. We supplemented the cellulases from *T. reesei* with purified PcβGlu1 and PcβGlu2 from *P. citrinum* UFV1 for hydrolysis of colloidal preparations of sugarcane bagasse, banana leaf, and coconut fiber with minimal pre-treatment [30]. The supplementation assessed the synergistic effect of *P. citrinum* UFV1 PcβGlu2 on *T. reesei* cellulases activities.

Abundantly available lignocellulosic crop residues as sugar cane bagasse, coconut fiber or banana pseudostem can be used in biorefineries for ethanol production. Some studies have shown that different biomasses have different chemical compositions [54, 55]. Hence, cellulolytic enzymes secreted by different organisms, and induced in liquid or solid medium, can act differently on these natural substrates. Specificities of the enzymes will determine the type of material they can act upon efficiently.

The colloidal biomasses used in this work use a minimum pre-treatment, and after drying milling, sieving, and wet milling, they are transformed into a brown and homogeneous suspension of fine particles with a broad distribution of size (mostly particles up to 5 μm in diameter) [30]. Thus, we believe that the small size of these particles may facilitate the action of cellulases. However, the low hydrolysis capacity of the enzymes on sugarcane bagasse may have been caused by a process of adsorption to the lignin present. To have a better understanding of this phenomenon, a more detailed characterization of the composition of the various sources used for hydrolysis and energy production would be necessary. These biomasses have different lignin compositions and non-specific binding of cellulases to lignin has been implicated as the main factor in the loss of cellulose activity during biomass conversion to sugars.

Thus, depending on the composition of the biomass and the specificity of the enzyme, these can interact in different ways, which leads to differences in hydrolysis ability. Specific enzyme adsorption to lignin from a mixture of biomass hydrolyzing enzymes is a competitive affinity and both hydrophobic and electrostatic interactions are responsible for this binding phenomenon [56] and lignin from different plant origins coupled with various pre-treatment chemistries might result in a variable adsorption surface chemistry and enzyme accessibility [57].

We demonstrated that PcβGlu2 is more efficient than Pcβglu1 in this kind of application. Pcβglu2 was able to hydrolyze substrates tested without the addition of any other enzyme and also supplemented *T. reesei* cocktail, increasing the final hydrolysis percentage of banana pseudostem. It is important to notice that, from the three biomass sources used, banana pseudostem has the lower content of lignin, around 9% [58], in comparison with sugarcane (17–24%) [59] and coconut (up to 42%) [60]. In this way, the observation of a better performance of Pcβglu2 when supplementing *T. reesei* enzymes in the hydrolysis of banana pseudostem, in comparison with the other sources of biomass tested, might be related to a lower lignin content. Surprisingly, *P. citrinum* enzymes showed a significant activity against coconut fiber, the biomass with the highest content of lignin among the tested, and when Pcβglu2 was used together with *T. reesei* cocktail, the final quantity of reducing sugars released from this material increased almost twice. Nevertheless, these data must be considered with caution, because the results in some cases suggest inhibition when the different enzymes sources are mixed (e.g., sugarcane bagasse in Fig. 9a, d) or an insignificant activation at the initial times of hydrolysis (coconut fiber in Fig. 9c, f).

As a whole, all these characteristics indicate that the enzymes secreted by *P. citrinum*, especially Pcβglu2, might be reliable candidates to improve the hydrolysis of specific biomass sources. As noted by *k*_cat_ values and subsite affinity mapping, these enzymes were capable of hydrolyzing a broad range of oligosaccharides with high catalytic efficiency. The concerted action of exo- and endo-cellulases, together with hemicellulases, might result in temporary accumulation of oligosaccharides with intermediate size, or even disaccharides as laminaribiose, which might inhibit cellulases. In this respect, as far as biorefinery process is concerned, it is interesting that the *β*-glucosidases used possess also specificity for di/oligosaccharides other than cellobiose because of the use of heterogeneous biomass feedstocks.

## Conclusions

*Penicillium citrinum* UFV1 produces two *β*-glucosidases when cultivated under SMF using wheat bran as a carbon source. These enzymes use a double displacement hydrolysis mechanism and might belong to GH3 family. The purified enzymes, PcβGlu1 and PcβGlu2, have different molecular and biochemical characteristics, at least five subsites for glycosyl residue binding in their active site, and have a broad substrate specificity. Both enzymes showed relevant features to be applied in biotechnological processes, such as biomass saccharification. PcβGlu2 showed an interesting potential by acting synergically with *T. reesei* cocktail in the hydrolysis of very complex and lignin-rich biomass, as colloidal banana pseudostem and coconut fiber.

## Methods

### Reagents

The reagents *p*-nitrophenyl *β*-d-glucopyranoside (ρNPβGlu), methylumbelliferyl-*β*-glucopyranoside (MUβGlu), octyl-*β*-glucopyranoside (octylβGlu), gentiobiose, laminaribiose, cellobiose, cellotriose, cellotetraose, cellopentaose, microcrystalline cellulose (Avicel^®^, Cat 11365), low viscosity carboxymethylcellulose (CMC, Cat C5678), *ρ*-nitrophenyl-*β*-d-galactopyranoside (ρNPβgal), methylumbeliferil-*β*-d-mannopyranoside (MUβMan), ρ-nitrophenol, 4-methylumbelliferone, cellulases from *T. reesei* ATCC 26921 (Cat. C8546) and the molecular markers Blue dextran (Cat. D4772), Cytochrome C (Cat. 12 kDa), carbonic anhydrase (Cat. C7025), serum albumin bovine (Cat. A8531), alcohol dehydrogenase (Cat. A8656), and beta amylase (Cat. A8781) were purchased from Sigma-Aldrich Company (St. Louis, Missouri, USA). Trypsin (Cat. V511A) for mass spectrometry was acquired from Promega Corporation (Madison, Wisconsin, USA). Glucose oxidase-based glucose quantitation reagent was acquired from Bioclin (Minas Gerais, Brazil). Other reagents used in this work were analytical grade.

### Organism and culture conditions

*Penicillium citrinum* UFV1 was obtained from the mycological collection of the Seed Pathology and Post Harvest Laboratory, Federal University of Viçosa, MG, Brazil. The fungus was maintained on potato dextrose agar (PDA) at 4 °C. To evaluate enzyme induction, fungus was maintained in PDA plates for 7 days at 28 °C for sporulation, and spores from this culture were inoculated into 1000 mL Erlenmeyer flasks containing 400 mL of minimal requirement (MR) medium at a concentration of 10^7^ spores mL^−1^ after sterilization. The MR medium consisted of 0.3% K_2_HPO_4_, 1.05% KH_2_PO_4_, 0.015% MgSO_4_·7H_2_O, 0.15% (w/v) (NH_4_)_2_SO_4_, 0.09% yeast extract, and 1% of wheat bran as carbon source [61]. The culture was incubated at 28 °C and 180 rpm for 240 h. The soluble fraction was separated by filtration through nylon cloth and centrifugation at 21,000×*g* for 15 min at 4 °C and used as a source of secreted enzymes.

#### Enzymatic assays and protein quantitation

Unless otherwise specified, *β*-glucosidase activities were assayed at 40 °C in 50 mM sodium acetate buffer, pH 5.0. Assays with substrates 0.5 mM *p*-nitrophenyl *β*-d-glucopyranoside (pNPβGlu), 0.5 mM *p*-nitrophenyl *β*-d-galactopyranoside (pNPβGal), 14 µM methylumbelliferyl-*β*-glucopyranoside (MUβGlu), or 14 µM methylumbelliferyl-*β*-mannopyranoside (MuβMan) were interrupted after different time intervals by adding 0.25 M Na_2_CO_3_. The amount of *p*-nitrophenol (pNP) released was determined at 410 nm and the amount of 4-methylumbelliferone released was determined at 355 nm excitation and 460 nm emission. Assays with 0.5 mM cellobiose, cellotriose, cellotetraose, cellopentaose, laminaribiose, gentiobiose, or octyl-*β*-glycopyranoside were interrupted at different time intervals by incubating the mixture at 99 °C for 5 min. The amount of glucose released was determined at 505 nm according to glucose oxidase method [62]. Assays against 0.25% laminarin, xylan, microcrystalline cellulose (Avicel^®^), or carboxymethylcellulose (CMC) were interrupted after different time intervals by incubating the mixture at 99 °C for 5 min in a thermocycler (Applied Biosystems, Singapore). Reducing sugars released were determined as previously described [63, 64]. Controls without enzyme or substrate were included. One unit of enzymatic activity (U) was defined as the amount of enzyme which released 1 μmol of product/min.

Protein concentrations were determined with the BCA method [65]; for samples containing ammonium sulfate, they were treated with deoxycholic acid previously to BCA assay [66]. For purified samples, protein concentration was determined according to the principle of silver binding [67].

### Purification of *β*-glucosidases

#### Hydrophobic interaction chromatography

Solid (NH_4_)_2_SO_4_ was added to the soluble fraction of *P. citrinum* liquid cultures (see “Organism and culture conditions” section) to achieve 50% saturation. After 30 min at 4 °C with mixing, sample was submitted to centrifugation at 21,000 *g* for 15 min at 4 °C; the precipitate was discarded and 5 mL of supernatant was applied to a HiTrap Phenyl FF column (2.5 cm × 1.6 cm) (AKTA Prime Plus, GE, Uppsala, Sweden) equilibrated in 50 mM sodium acetate pH 5 containing (NH_4_)_2_SO_4_ 2.2 M (Buffer A). Elution was undertaken with 2.2–0 M (NH_4_)_2_SO_4_ gradient by mixing Buffer A with increasing amounts of 50 mM sodium acetate pH 5, at 2 mL min^−1^. Fractions of 5 mL each were collected and assayed using MUβGlu and pNPβGlu substrates. The more active fractions (12–16, named PcβGlu1 and 25–30, named PcβGlu2) were pooled and stored at − 20 °C for further analysis.

#### Gel filtration chromatography

Pooled fractions corresponding to PcβGlu2 in the “Hydrophobic interaction chromatography” were applied (0.5 mL) onto a Superdex 200 10/300 GL (1.0 cm × 30 cm) (AKTA Purifier, GE, Uppsala, Sweden) equilibrated in 50 mM sodium acetate pH 5 containing 100 mM (NH_4_)_2_SO_4_ and eluted with the same buffer at 0.5 mL min^−1^. Fractions of 0.5 mL were collected and assayed using MUβGlu substrate. The more active fractions (29–32) were pooled, BSA 1 mg mL^−1^ was added for stabilization and this material was stored at 4 °C until analysis.

### SDS-PAGE, PAGE, and relative molecular mass measurements

Samples were precipitated with TCA [68] and SDS-PAGE (7.5%, w/v) analysis of enzyme fractions was performed according to the method of Laemmli and gels were stained with silver nitrate [69, 70].

For nondenaturing polyacrylamide gel electrophoresis (PAGE), samples were desalted by dialysis against 10 mM sodium acetate buffer pH 5.0 and concentrated in Centricon with 30 kDa cutoff (Millipore Corporation, Massachusetts, USA). *β*-Glucosidase activity was detected on the polyacrylamide gel with MUβGlu 4 mM in sodium acetate buffer [71]. Methylumbelliferone released from MUβGlu was visualized under ultraviolet light.

The apparent native molecular mass of the purified *β*-glucosidases was estimated using a Superdex 200 10/300 GL (1.0 cm × 30 cm) (GE, Uppsala, Sweden). The column was equilibrated and eluted with 50 mM sodium acetate pH 5 at 0.5 mL min^−1^. The void volume was determined using Blue dextran (2000 kDa). Cytochrome C (12 kDa), carbonic anhydrase (29 kDa), serum albumin bovine (66 kDa), alcohol dehydrogenase (150 kDa), and beta amylase (200 kDa) were used as molecular mass standards.

### Protein identification by mass spectrometry

PcβGlu1 and PcβGlu2 samples were dialyzed against 10 mM sodium acetate buffer pH 5 and concentrated in Concentrator 5301 (Eppendorf, Hamburg, Germany). Desalted enzymes were reduced, alkylated, and subjected to trypsin digestion for 24 h at 37 °C, 1:50 enzyme:substrate (m:m) [72]. Tryptic peptides were desalted with ZipTip C_18_ (Millipore Corporation, Massachusetts, USA) according to manufacturer instructions. Samples were dried in Concentrator 5301 (Eppendorf, Hamburg, Germany), ressuspended in formic acid 1%, and submitted to MS/MS analysis using an EASY-nLC coupled to a nanoESI LTQ Orbitrap XL instrument (Thermo Scientific, USA). Chromatographic and mass spectrometric analyses’ conditions were the same as previously described [73], although the separation column was only 12-cm long and gradient conditions were: 2–60% B during 52 min; up to 80% B in 4 min, maintaining at this concentration for 2 min more. All MS/MS spectra were analyzed using PEAKS Studio 8.5 build 20180105 (Bioinformatics Solutions, Canada). After data refinement with the precursor (mass only) correction option, PEAKS DE NOVO analysis was run assuming trypsin digestion, with a fragment ion mass tolerance of 0.60 Da and a parent ion tolerance of 15 ppm. Cysteine (C) carbamidomethylation (+ 57.02 Da) was set as fixed modification and the following variable modifications were searched: deamidation at N/Q (+ 0.98 Da), oxidation at M and unspecific carbamidomethylation (+ 57.02) at D, E, H, K, and free N-terminus; a maximum of 2 variable modifications per peptide was allowed. PEAKS DB analysis was performed using these same parameters plus the possibility of up to two missed enzyme cleavages and non-specific cleavage at one side of the peptides. Searches were made against a subdatabase, composed of all Uniprot entries that matched the term “Penicillium” (338,849 entries, downloaded on April 18, 2018). Finally, 310 other possible modifications were searched with the PEAKS PTM algorithm, using the same parameters described above, against a protein subdatabase composed only by protein entries found by the joint PEAKS DE NOVO and PEAKS DB searches. False discovery rates (FDR) were estimated through the PEAKS decoy fusion approach. A peptide-spectrum match FDR of 0.1% and protein identifications with at least 2 unique peptides were the criteria used to establish FDR values at peptide and protein levels ≤ 1%.

### Configuration of the anomeric carbon atom of reaction products

To identify the anomeric configuration of the d-glucose released by *β*-glucosidases [74], enzymes were incubated at 40 °C in 50 mM sodium acetate buffer pH 5 with cellobiose as a substrate. After 30 min for PcβGlu1 and 120 min for PcβGlu2, reactions were stopped by the addition of 100 mM glucono-*δ*-lactone to the incubation medium, and aliquots of 0.1 mL were removed. Each sample’s aliquot was incubated at 100 °C for 5 min followed by addition of 0.1 mL of glucose oxidase reagent (Bioclin, Minas Gerais, Brazil). The absorbance was recorded continuously at 505 nm for 20 min. Controls were prepared similar to the experimental samples and the substrate was replaced by a fresh solution of α-d-glucose or by a solution of α-d-glucose incubated at 100 °C for 5 min.

### Kinetic studies

The effect of pH on enzyme stability and activity was determined at 40 °C using the following buffers (100 mM): for PcβGlu1, citrate/phosphate (pH 3–7), EPPS (pH 7–9), AMPSO (pH 9–10), CAPS (pH 10–11) and for PcβGlu2, sodium acetate (pH 3.7–6), MOPS (pH 6–8), EPPS (pH 7–9), and AMPSO (pH 9–10). For pH stability assays, enzymes were pre-incubated in the determined pH for 4 h at 40 °C to residual activity determination according to “Enzymatic Assays and Protein Quantitation” section MUβGlu as a substrate.

Thermostability was evaluated by incubating PcβGlu1 in the presence of ammonium sulfate and PcβGlu2 in the presence of BSA 1 mg mL^−1^ for 4 h at temperatures from 30 to 80 °C. PcβGlu1 was also incubated at 50 °C in the absence of ammonium sulfate and PcβGlu2 incubated at 50 °C in the absence of BSA. The residual activities were determined according to “Enzymatic Assays and Protein Quantitation” section using MUβGlu as a substrate. Half-life (t_1/2_) and inactivation constant (*k*_*d*_) were calculated from a linear plot of log (residual activity) versus incubation time [75].

### Effect of metal ions

The effect of NaCl, NaNO_3_, KCl, NH_4_Cl, Pb(NO_3_)_2_, BaCl_2_, ZnCl_2_, MgCl_2_, MnCl_2_, CaCl_2_, CoCl_2_, FeCl_3_, HgCl, and CuSO_4_ on PcβGlu1 and PcβGlu2 activity was determined by pre-incubating enzymes for 30 min at 40 °C in 50 mM sodium acetate buffer pH 5 in the presence of 100 mM of each salt above. Residual activities were determined as described above using MUβGlu as a substrate.

### Determination of kinetic parameters

**Scheme 1:**
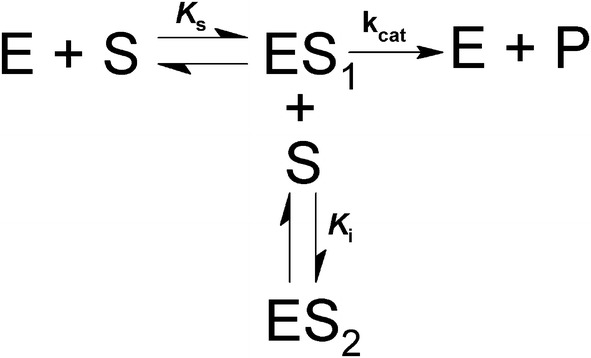
Model representation of a simple competitive substrate inhibition. According to this, at high concentrations, a second molecule of substrate (S) binds to the enzyme–substrate complex (ES_1_), forming an inactive ternary complex (ES_2_)

The values of the Michaelis constant (*K*_m_) and the maximum velocity (*V*_max_) were determined for PcβGlu1 and PcβGlu2 incubating enzymes in 50 mM sodium acetate buffer pH 5.0 at 40 °C with pNPβGlu, MUβGlu, OctylβGlu, gentiobiose, laminaribiose, cellobiose, cellotriose, cellotetraose, cellopentaose, or laminarin at concentrations ranging from 0.04 to 20 mM depending on substrate. Inhibition of PcβGlu1 and PcβGlu2 by glucose (0–15 mM) and cellobiose (0–10 mM) was determined in the presence of pNPβGlu as the substrate. Activity was determined depending on the substrate as described above. Values for *K*_m_, *V*_max,_ and *K*_i_ were determined using the GraFit software (GraFit version_7.0.3, Erithacus Software Limited) and Michaelis–Menten equation [75]. When high substrate concentration inhibition was detected, kinetic parameters were calculated taking into account the reaction below (Scheme 1) [76]:

1 $$V = \frac{{V_{\text{max} } \left[ S \right]}}{{K_{m } + \left[ S \right] \left( {1 + \frac{\left[ S \right]}{{K_{i} }}} \right)}}.$$

### Subsite mapping

The subsite affinities of *β*-glucosidases were evaluated according to the Hiromi’s method [29]. Subsite affinities *A*_*i*_ (kcal mol^−1^) for glucose and *k*_int_ (catalytic rate independent of the polymerization degree of the substrate) were calculated from *K*_m_ values and catalytic rate constants (*k*_cat_) for the hydrolysis of cellodextrins with polymerization degrees from 2 to 5. The validity of the calculations was subsequently confirmed by comparing experimental and theoretical *k*_cat_*, K*_m,_ and *k*_cat_*/K*_m_ values following Hiromi’s method to estimate the validity of *A*_*i*_ and *k*_int_ values obtained with errors of less than 20%. A2 was calculated by the method II. The theoretical values were obtained according to the equations:

2 $$K_{n,1 } = 0.021\exp \left( {\mathop \sum \limits_{i = 1}^{n} \frac{{A_{i} }}{\text{RT}}} \right)$$

3 $$K_{n,2} = 0.021\exp \left( {\mathop \sum \limits_{i = 2}^{n + 1} \frac{{A_{i} }}{\text{RT}}} \right)$$

4 $$\left( {\frac{I}{{K_{m} }}} \right)_{n } = K_{n,1 } + K_{n,2}$$

5 $$(k_{\text{cat}} )_{n} = k_{{\text{int} }} \left( {\frac{{K_{n,1 } }}{{K_{n,1 } + K_{n,2} }}} \right)$$

6 $$\left( {\frac{{k_{\text{cat}} }}{{K_{m} }}} \right)_{n } = k_{{\text{int} }} 0.021\exp \left( {\mathop \sum \limits_{i = 1}^{n} \frac{{A_{i} }}{\text{RT}}} \right),$$

where *n* is the substrate degree of polymerization, 0.021 corresponds to the contribution of the mixing entropy in water at 40 °C [29], *R* is the gas constant, and *T* is the absolute temperature.

### Biomass saccharification

*Penicillium citrinum* UFV1 purified *β*-glucosidases (PcβGlu1 and PcβGlu2) and commercial cellulase from *T. reesei* ATCC 26921 were applied in saccharification experiments using different sources of biomass. Colloidal suspensions of sugar cane bagasse, coconut fiber, and banana pseudostem were prepared with a minimum pre-treatment [30]. PcβGlu1 and PcβGlu2 were concentrated in Centricon 30 (Millipore, Billerica, MA, USA) membrane filter. Enzymatic saccharification of colloidal biomasses was performed at an initial solid concentration of 2.5% (w/v) in 50 mM sodium acetate buffer at pH 5. Enzyme loading was 10 CBU/g biomass of PcβGlu1 or PcβGlu2 and 7 CBU/g biomass for *T. reesei* cellulase. Sodium azide 0.01% (w/v) was added to the reaction mixture to inhibit microbial growth. The reaction was carried out in an orbital shaker at 150 rpm and 40 °C for 24 h. Samples (0.5 mL) were taken from the reaction mixture at different time intervals, immediately heated to 100 °C to denature enzymes, cooled, and then centrifuged for 5 min at 21,000 *g*. The released reducing sugars in supernatant and total sugar at colloidal biomass were detected by the previous described methods [77, 78]. The saccharification hydrolysis rate was estimated comparing the reducing sugar released at saccharification with the quantity of total sugars at colloidal biomass. Statistical analysis graphs and data analysis were performed with the software GraphPad Prism version 5.01 for Windows. Two-way ANOVA was used followed by Tukey’s multiple comparison tests, *p* < 0.05.

## Additional files

**Additional file 1: Table S1.** Production of cellulases and hemicellulases by *Penicillium citrinum* cultivated in liquid media using wheat bran as carbon source. Activities are presented as U/mL ± SD. CMC—carboxymethylcellulose. Superscript numbers represent the number of days in culture to achieve the maximum production of each enzyme.

**Additional file 2: Table S2.** Effect of solutes on PcβGlu2 stability.

**Additional file 3: Table S3.** Summary of protein groups identified following mass spectrometry analysis of purified PcBglu1. GH—match to Glycoside Hydrolase Family (E.C.3.2.1.21).

**Additional file 4: Table S4.** Summary of protein groups identified following mass spectrometry analysis of partially purified PcBglu2. GH—match to Glycoside Hydrolase Family (E.C.3.2.1.21).

**Additional file 5: Table S5.** Comparison between experimental and theoric rate parameters for PcβGlu1 and PcβGlu2 of *P. citrinum.*

## Abbreviations

PcβGlu1: *Penicillium citrinum* UFV1 *β*-glucosidase 1
PcβGlu2: *Penicillium citrinum* UFV1 *β*-glucosidase 2
pNPβGlu: *p*-nitrophenyl *β*-d-glucopyranoside
pNPβGal: *p*-nitrophenyl *β*-d-galactopyranoside
MUβGlu: methylumbelliferyl-*β*-glucopyranoside
MuβMan: methylumbelliferyl-*β*-manopyranoside
OctylβGlu: octyl-*β*-glucopyranoside
CMC: carboxymethylcellulose
EPPS: 4-(2-hydroxyethyl)-1-piperazinepropanesulfonic acid
AMPSO: *N*-(1,1-dimethyl-2-hydroxyethyl)-3-amino-2-hydroxypropanesulfonic acid
CAPS: 3-(cyclohexylamino)-1-propanesulfonic acid
MOPS: 4 morpholinepropanesulfonic
TCA: trichloroacetic acid
BCA: bicinchoninic acid
BSA: Bovine Serum Albumin
